# Inferring potential small molecule–miRNA association based on triple layer heterogeneous network

**DOI:** 10.1186/s13321-018-0284-9

**Published:** 2018-06-26

**Authors:** Jia Qu, Xing Chen, Ya-Zhou Sun, Jian-Qiang Li, Zhong Ming

**Affiliations:** 1 School of Information and Control Engineering, China University of Mining and Technology, Xuzhou, 221116 China; 2 National Engineering Laboratory for Big Data System Computing Technology, Shenzhen University, Shenzhen, 518060 China; 3 College of Computer Science and Software Engineering, Shenzhen University, Shenzhen, 518060 China

**Keywords:** microRNA, Small molecule, Association prediction, Triple layer heterogeneous network

## Abstract

Recently, many biological experiments have indicated that microRNAs (miRNAs) are a newly discovered small molecule (SM) drug targets that play an important role in the development and progression of human complex diseases. More and more computational models have been developed to identify potential associations between SMs and target miRNAs, which would be a great help for disease therapy and clinical applications for known drugs in the field of medical research. In this study, we proposed a computational model of triple layer heterogeneous network based small molecule–MiRNA association prediction (TLHNSMMA) to uncover potential SM–miRNA associations by integrating integrated SM similarity, integrated miRNA similarity, integrated disease similarity, experimentally verified SM–miRNA associations and miRNA–disease associations into a heterogeneous graph. To evaluate the performance of TLHNSMMA, we implemented global and two types of local leave-one-out cross validation as well as fivefold cross validation to compare TLHNSMMA with one previous classical computational model (SMiR-NBI). As a result, for Dataset 1, TLHNSMMA obtained the AUCs of 0.9859, 0.9845, 0.7645 and 0.9851 ± 0.0012, respectively; for Dataset 2, the AUCs are in turn 0.8149, 0.8244, 0.6057 and 0.8168 ± 0.0022. As the result of case studies shown, among the top 10, 20 and 50 potential SM-related miRNAs, there were 2, 7 and 14 SM–miRNA associations confirmed by experiments, respectively. Therefore, TLHNSMMA could be effectively applied to the prediction of SM–miRNA associations.

**Electronic supplementary material:**

The online version of this article (10.1186/s13321-018-0284-9) contains supplementary material, which is available to authorized users.

## Background

MicroRNA (miRNA) is a small non-coding RNA molecule (about 22 nucleotides) discovered in plants, animals, human beings and even some viruses, that functions in RNA silencing and post-transcriptional regulation of gene expression [1, 2]. The first miRNA was discovered in the early 1990s [3, 4]. However, miRNAs were not recognized as a distinct class of biological regulators until the early 2000s [5, 6]. MiRNA research revealed multiple roles for miRNAs in many important biological processes [7–11]. MiRNAs function via base-pairing with complementary sequences within mRNA molecules, which results in these mRNA molecules silenced [12, 13]. Furthermore, aberrant miRNA expressions are implicated in various disease states [14–16], and miRNA-based therapies are under investigation [17]. Many studies have been conducted for the detection or regulation of miRNAs with bio-medical implications [18–20]. Regulation of miRNAs by synthesized oligonucleotides or small molecules is an efficient means to modulate endogenous miRNA function and treat miRNA-related diseases. They are being considered as a novel type of bio-markers or potential therapeutic targets for various diseases [21].

In molecular biology and pharmacology, a small molecule is a low molecular weight (< 900 Daltons) organic compound that may help regulate a biological process, with a size on the order of 1 nm [22]. Most drugs are small molecules. Small molecule regulators can modulate the regulatory networks of target miRNAs, and have potential use as probes to identify unknown components of miRNA pathways [23]. Regulation of oncogenic or tumor-suppressive miRNAs by small molecules can induce cancer cell apoptosis [24]. Several small molecules with different regulatory activities on miRNAs have been identified,including inhibitors of miR-21 and inhibitors and activators of miR-122 [25]. MiR-21 is a well-known oncogenic miRNA and the expression of which is extremely high in ovarian, breast, and lung cancers [26]. Regulation of miR-21 using small molecules may be a novel approach to cancer treatment. Streptomycin was identified as a specific inhibitor of miR-21 [27]. Thermal melting results indicated that the inhibitory activity of streptomycin was derived from its direct interaction with pre-miR-21 [27]. The decreased expression of miR-122 and over-expression of miR-122 in liver cancer cells can induce cancer cell apoptosis [28]. In addition, miR-122 could also promote the replication of the hepatitis C virus (HCV) [29]. Using dual-luciferase reporter gene, where the Renilla luciferase gene is regulated by miR-122, two small molecules were identified as specific inhibitors of miR-122, while another compound was a specific activator. They all targeted miR-122 transcription [30]. MiR-34a is a tumor-suppressive miRNA that is down-regulated in most cancers and targets several anti-apoptotic genes [31–33]. Up-regulation of miR-34a can cause cellular apoptosis and inhibit cellular differentiation [34]. MiR-34a mimics with the ability to restore the expression of miR-34a have been examined in clinical trials [34]. Using a hepatocellular carcinoma cell line, a small molecule was identified from a natural product library as a specific activator of miR-34a [35]. QRT-PCR analysis showed that both mature and primary miR-34a was up-regulated by this compound, indicating that it activated miR-34a at the transcriptional level [35].

Currently, a wide number of studies have been devoted to develop high-throughput methods to screen small molecule modifiers of miRNAs, which may provide a new direction for miRNA-targeting therapies [36]. MiRNA regulation by small molecules could result from inference in miRNA biogenesis at three levels: before, during and after transcription [37]. Small molecules increase or decrease miRNA expressions indirectly, by altering miRNA promoter regions or binding to the transcription factors [37]. They also can disrupt the maturation of miRNAs by binding with essential RNA-endonucleases [38]. In summary, investigating the relationships between small molecules and miRNAs is important for disease therapy and clinical applications for known drugs [36, 37]. However, it is time-consuming to identify the regulations between small molecules and miRNAs by experimental approaches owing to the high complexity of biological systems. Therefore, there is an urgent need to develop new computational approaches or models to decipher the relationships between small molecules and miRNAs to speed up pharmacy genomic studies.

Some computational methods have been established to comprehensively identify the potential associations between SMs and miRNAs depending on the assumption that similar SMs are more likely to have associations with similar miRNAs. For example, Li et al. [39] proposed a miRNA pharmacogenomic framework of small molecule–MiRNA network-based inference (SMiR-NBI) model, in which they constructed a heterogeneous network connecting drugs, miRNAs as well as genes and implemented network based inference (NBI) on the network to identify the underlying mechanisms of anticancer drug responses mediated by miRNAs. The model with high prediction accuracy and low computation cost only takes advantage of the network topology information from the built network as input. Lv et al. [40] constructed an heterogeneous molecular network to successfully identify novel SM-related miRNA targets based on the integration of SM side effect similarity, SM chemical structure similarity, gene functional consistency-based similarity for SMs and miRNAs, disease phenotype-based similarity for miRNAs and SMs, known miRNA–SM associations using a similarity-based random walk with restart. Furthermore, Jiang et al. [41] introduced a novel computational method to discover potential miRNA–SM associations in 23 different cancers on the basic of differential expression of miRNA target genes and gene signatures that are extracted from the gene expression profiles following drug treatment of the 23 cancers. As a result, they built the small molecule-miRNA network (SMirN) for 17 different cancers and identified miRNA modules and SM modules in each of the cancer specific SMirNs. Using the constructed network and identified modules, they predicted new miRNAs for drug target and drug candidates for cancer therapy. Wang et al. [42] presented a novel model to successfully predict potential miRNA–SM associations based on miRNA and SM functional similarity network, in which they calculated functional similarity for each pair of SM and miRNA based on Gene Ontology (GO) annotations of miRNA perturbed gene expression profiles and SM perturbed gene expression profiles. It is worth noting that potential drugs-diseases associations could be predicted at the same time through combining known miRNA–SM associations with experimentally validated miRNA–disease associations, which would be helpful for drug repositioning. Recently, Meng et al. [43] built a bioactive Small molecule and miRNA association network in Alzheimer’s Disease (SmiRN-AD) to predict novel miRNA–SM associations based on the gene expression signatures of bioactive SM perturbation and miRNA regulation. Furthermore, the topological characteristics and functional properties of miRNAs and SMs were comprehensively analyzed in SmiRN-AD. Lastly, they constructed a database for SmiRN-AD and differential expression patterns of AD-associated miRNA targets can also be provided. Thus, the method and its application may be help for providing a new view with respect to the treatment of AD. Currently, in large-scale studies, high-performance or high-precision computing approaches are still required to comprehensively identify the potential miRNA–SM associations.

In this study, we developed an effective computational method of triple layer heterogeneous network based small molecule-MiRNA association prediction (TLHNSMMA) by combining integrated SM similarity, integrated miRNA similarity, integrated disease similarity, experimentally verified miRNA–SM associations and miRNA–disease associations into a triple layer heterogeneous network. An iterative updating algorithm that propagates information across the constructed heterogeneous network is then developed to predict novel associations between SMs and miRNAs. Moreover, new miRNA–disease associations can be automatically established at the same time. In this model, the known miRNA–SM associations were download form the database of SM2miR v1.0 [44]. We constructed two groups of datasets based on the known miRNA–SM association and employed TLHNSMMA to predict new miRNA–SM associations based on the two datasets respectively. In the Dataset 1, only a part of SMs and miRNAs were involved in the known miRNA–SM associations. In Dataset 2, all the SMs and miRNAs are implicated in the known miRNA–SM associations. To evaluate the effectiveness of TLHNSMMA, global and local leave-one-out cross validation (LOOCV) as well as fivefold cross validation were implemented. In short, The AUCs of global LOOCV are 0.9859 and 0.8149 for Dataset 1 and Dataset 2, respectively; the AUCs of local LOOCV by fixing each miRNA to predict miRNA-associated SMs are respectively 0.9845, 0.8244 for the two datasets; the AUCs of local LOOCV by fixing each SM to predict SM-associated miRNAs are respectively 0.7645, 0.6057 for the two datasets. For fivefold cross validation, the average AUCs and standard deviations are 0.9851 ± 0.0012, 0.8168 ± 0.0022 for the two datasets, respectively. In case studies, 2 out of the top 10 and 14 out of the top 50 predicted miRNA–SM associations were confirmed by published references. Therefore, it proves that TLHNSMMA is effective in predicting potential associations between miRNAs and SMs.

## Results

### Performance evaluation

We used global and local LOOCV as well as fivefold cross validation based on the known SM–miRNA associations in SM2miR v1.0 database to evaluate the performance of TLHNSMMA. Meanwhile, TLHNSMMA was compared with one previous classical computational methods: SMiR–NBI [39] in cross validation. SMiR–NBI only rely on known miRNA–SM associations [39]. The known miRNA–SM association dataset used for this comparison was the same as that in our study, i.e., the 664 known associations between 831 miRNAs and 541 diseases (Dataset 1) and the known 664 known associations between 39 SMs and 286 miRNAs (Dataset 2). The SMiR-NBI model was constructed based on the state-of-the-art network-based inference (NBI) algorithm [45, 46]. For initial resources of a given SM located in its regulated miRNAs. Each miRNA will distribute resources equally to all neighboring SMs and then redistribute their obtained resources to every adjacent miRNA. The final resources score for miRNAs represented their potential association to be regulated by the interested SM [46].

In LOOCV, each known miRNA–SM association in the dataset was alternately used as the test sample in turn, while other known miRNA–SM associations were considered as training samples. The miRNA–SM without known association were regarded as candidate samples. After TLHNSMMA was implemented, we would obtain the prediction scores of each miRNA–SM pair. In global LOOCV evaluation, the score of test sample would be compared with the scores of all the candidate samples. However, In the SM-fixed local LOOCV, the test sample would be ranked with the scores of the candidate samples which composed of all the miRNAs that do not associated with the fixed SM. In the miRNA-fixed local LOOCV, the test sample would be ranked with the scores of the candidate samples composed of all the SMs without any known associations with the fixed miRNA. In fivefold cross validation, all the experimentally verified miRNA–SM associations were randomly divided into five equal groups. Each time, four groups were selected as training samples in turn and the other one group would be considered as test sample. Similarly, the miRNA–SM pairs with no known associations were regarded as candidate samples. Then, the score of each test sample would also be compared with that of all the candidate samples, respectively. The procedure of fivefold cross validation would be repeated 100 times in this model.

**Fig. 1:**
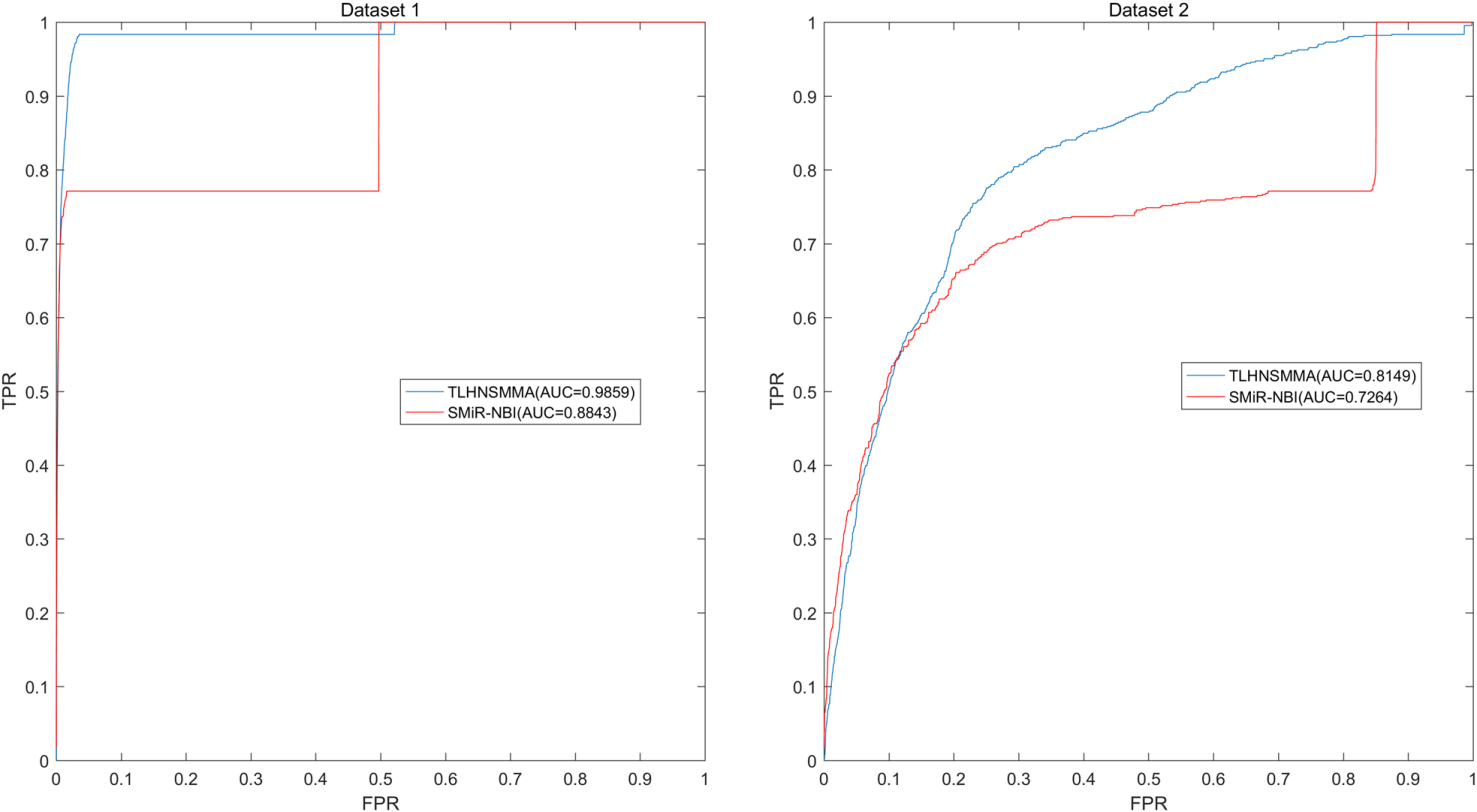
Performance evaluation comparison between TLHNSMMA and SMiR–NBI in terms of ROC curve and AUC based on global LOOCV in Dataset 1 (left) and Dataset 2 (right). As a result, TLHNSMMA achieved AUCs of 0.9859 and 0.8149 for Dataset 1 and Dataset 2, respectively. The predictive performance of TLHNSMMA is better than SMiR–NBI

**Fig. 2:**
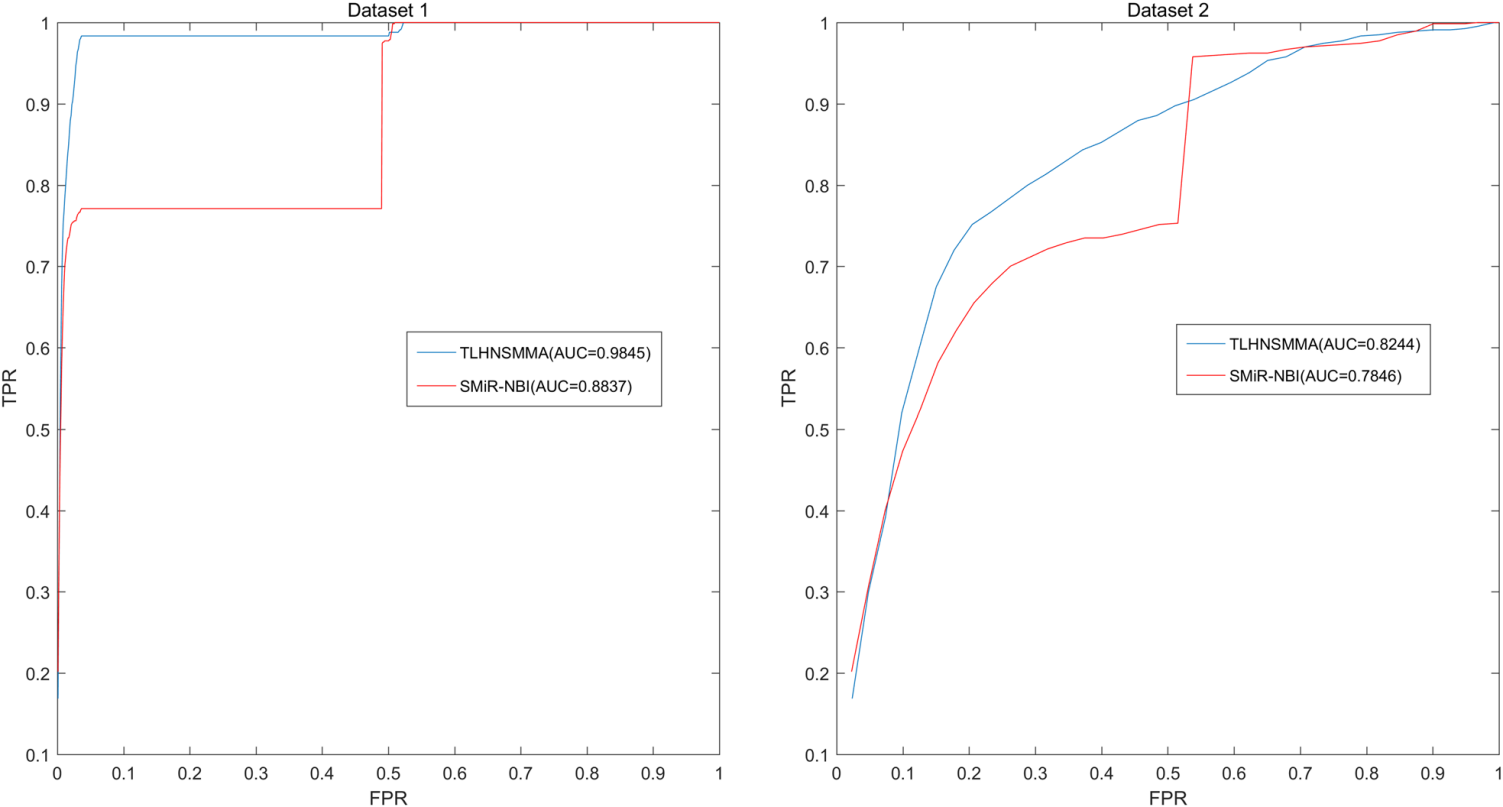
Performance evaluation comparison between TLHNSMMA and SMiR–NBI in terms of ROC curve and AUC based on local LOOCV by fixing miRNAs to rank SMs in Database 1 (left) and Database 2 (right). As a result, TLHNSMMA achieved AUCs of 0.9845 and 0.8244 for Dataset 1 and Dataset 2, respectively. The predictive performance of TLHNSMMA is better than SMiR-NBI

**Fig. 3:**
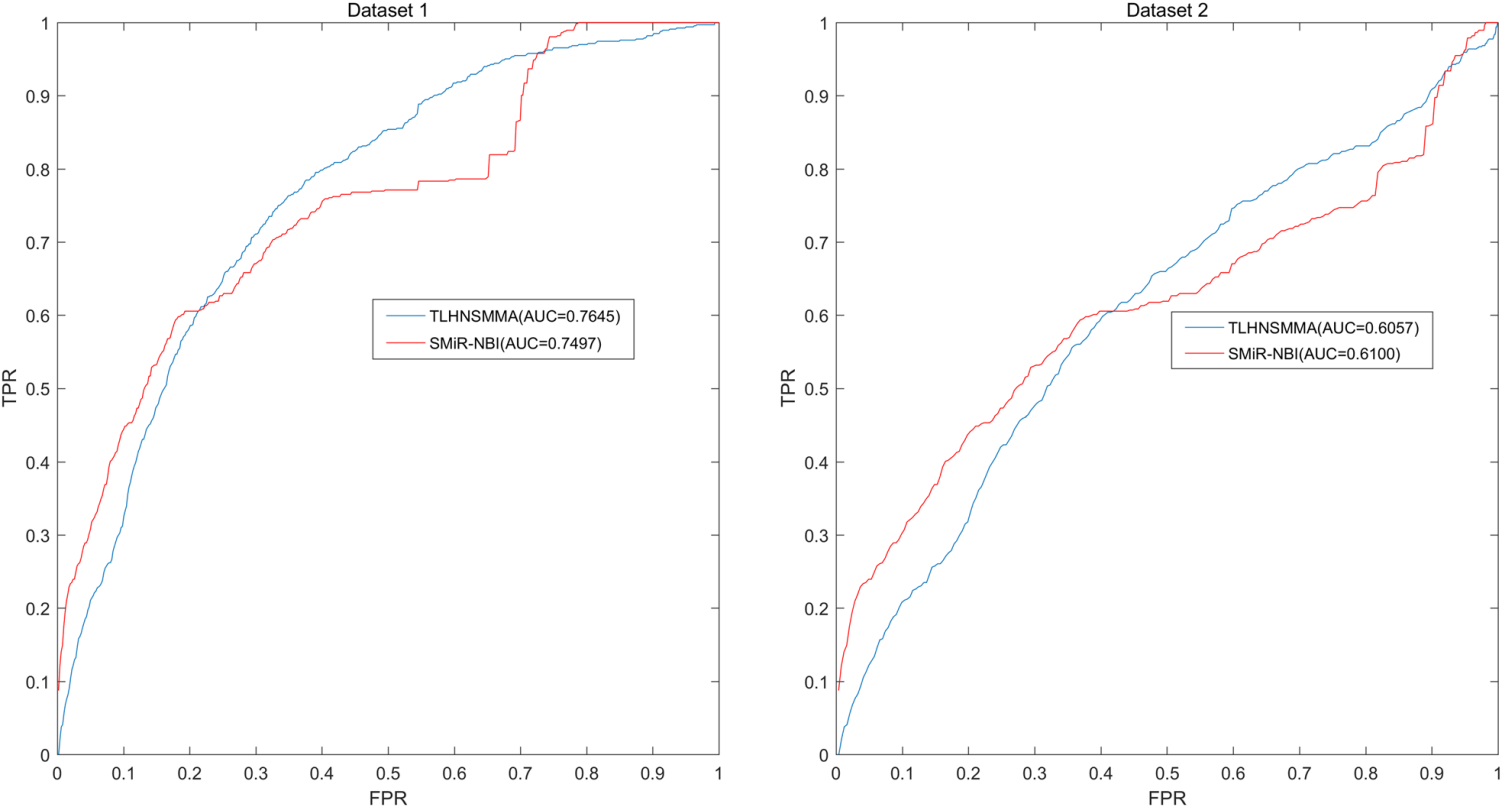
Performance evaluation comparison between TLHNSMMA and SMiR–NBI in terms of ROC curve and AUC based on local LOOCV by fixing SMs to rankmiRNAs in Database 1 (left) and Database 2 (right). As a result, TLHNSMMA achieved AUCs of 0.7645 and 0.6057 for Dataset 1 and Dataset 2, respectively. The predictive performance of TLHNSMMA is better than SMiR–NBI

Finally, we plotted Receiver operating characteristics (ROC) curve using true positive rate (TPR, sensitivity) against the false positive rate (FPR, 1-specificity) at different thresholds. Sensitivity denotes the percentage of positive miRNA–SM pairs that are correctly identified among all positive miRNA–SM pairs. Meanwhile, specificity refers to the percentage of negative miRNA–SM pairs that are correctly predicted among all negative miRNA–SM pairs. Area under the ROC curve (AUC) was calculated as a form of evaluation index for the model. In the model, the higher the AUC value, the better the prediction ability. When the model has perfect prediction ability, the value of AUC is 1. Meanwhile, if the model only possesses random prediction ability, the value of AUC is 0.5. As a result, in global LOOCV, TLHNSMMA and SMiR-NBI obtained AUCs of 0.9859 and 0.8843 based on Dataset 1, respectively. TLHNSMMA and SMiR-NBI obtained AUCs of 0.8149 and 0.726 based on Dataset 2, respectively (see Fig. 1). In the framework of miRNA-fixed local LOOCV, the AUCs of TLHNSMMA and SMiR-NBI based on Dataset 1 are 0.9845 and 0.8837, respectively. In addition, the AUCs of TLHNSMMA and SMiR-NBI based on Dataset 2 are 0.8244 and 0.7846, respectively (see Fig. 2). In the framework of SM-fixed local LOOCV, The AUCs of TLHNSMMA and SMiR-NBI based on Dataset 1 are 0.7645 and 0.7497, respectively. Furthermore, the AUCs of TLHNSMMA and SMiR-NBI based on Dataset 2 are 0.6057 and 0.6100, respectively (see Fig. 3). In fivefold cross validation, TLHNSMMA and SMiR-NBI obtained AUCs of 0.9851 ± 0.0012 and 0.8554 ± 0.0063 based on Dataset 1, Meanwhile, TLHNSMMA and SMiR-NBI obtained AUCs of 0.8168 ± 0.0022 and 0.7104 ± 0.0087 based on Dataset 2. Finally, in order to obtain a clear knowledge of the predictability performance of TLHNSMMA compared with SMiR-NBI in our study. We listed evaluation result of TLHNSMMA and SMiR-NBI in global LOOCV, SM-fixed local LOOCV, miRNA-fixed local LOOCV and fivefold cross validation (see Table 1). In general, TLHNSMMA turns out to be more reliable and effective in predicting potential miRNA–SM associations compared with SMiR-NBI.

**Table 1:** Performance evaluation comparison between TLHNSMMA and SMiR-NBI in global LOOCV, SM-fixed local LOOCV, miRNA-fixed local LOOCV and fivefold cross validation based on Dataset 1 and Dataset 2

Dataset	Experimental types	TLHNSMMA	SMiR-NBI
Dataset 1	AUC in global LOOCV	0.9859	0.8843
	AUC in SM-fixed local LOOCV	0.7645	0.7497
	AUC in miRNA-fixed local LOOCV	0.9845	0.8837
	Average AUC in fivefold cross validation	0.9851 ± 0.0012	0.8554 ± 0.0063
Dataset 2	AUC in global LOOCV	0.8149	0.7264
	AUC in SM-fixed local LOOCV	0.6057	0.6100
	AUC in miRNA-fixed local LOOCV	0.8244	0.7846
	Average AUC in fivefold cross validation	0.8168 ± 0.0022	0.7104 ± 0.0087

The corresponding AUCs of TLHNSMMA are shown in the third columns, and compared with the AUCs for SMiR–NBI in the fourth column

In addition, in order to assess the baseline performance of TLHNSMMA based on the dataset of known miRNA–SM associations to see whether or not the dataset of known miRNA–SM associations exist false positives. We removed all known miRNA–disease associations in the dataset, and randomly selected 664 miRNA–SM pairs from all miRNA–SM pairs as known associations. Then we implemented TLHNSMMA on the new randomly created adjacency matrix to calculate the AUC value for global LOOCV, SM-fixed local LOOCV and miRNA-fixed local LOOCV based on Dataset 1 and Dataset 2, respectively. We repeat 100 times for each process mentioned above. More importantly, if some false positives exist in the dataset of known miRNA–SM associations, the output of TLHNSMMA will be better than random prediction. On the other hand, if there are almost no false positives exist in the dataset of known miRNA–SM associations, the performance of TLHNSMMA will be similar to the random prediction. Therefore, we implemented the hypothesis testing that the six results of LOOCV mentioned above equal to the random performance, i.e. with random AUC of 0.5, respectively. We implemented *t* test on the results of LOOCV to assess the significance of hypothesis testing. Based on Dataset 1, we obtained the p value of 0.7757 (in global LOOCV), 0.5704 (in SM-fixed local LOOCV) and 0.0825 (in miRNA-fixed local LOOCV), Based on dataset 2, we obtained the p-value of 0.2612 (in global LOOCV), 0.6979 (in SM-fixed local LOOCV) and 0.6910 (in miRNA-fixed local LOOCV). The results shown that the p value calculated are all higher than 0.05, indicating that the performance of TLHNSMMA will be similar to the random prediction and hence there are almost no false positives exist in the dataset of known miRNA–SM associations.

## Case studies

Based on the published references in PubMed database, we verified the prediction results of TLHNSMMA. Through the case studies, we can further confirm the effectiveness of the TLHNSMMA. We ulteriorly observed the number of the verified miRNA–SM associations in the top 10, top 20 and top 50 ones predicted by the computational model. As the result shown, among the top 10, 20 and 50 potential small molecule–miRNA associations, there were 2, 7 and 14 associations confirmed by experiments, respectively (see Table 2).

**Table 2:** Verification of the top 50 predicted miRNAs associated with SMs based on published references

SM	MiRNA	Evidence	SM	MiRNA	Evidence
CID:3385	hsa-mir-219-a	Unconfirmed	CID:5757	hsa-mir-125b-1	Unconfirmed
CID:448537	hsa-mir-219-a	Unconfirmed	CID:448537	hsa-mir-125b-2	Unconfirmed
CID:5757	hsa-mir-219-a	Unconfirmed	CID:3385	hsa-mir-29b-1	Unconfirmed
CID:5311	hsa-mir-219-a	Unconfirmed	CID:448537	hsa-mir-145	Unconfirmed
CID:3229	hsa-mir-219-a	Unconfirmed	CID:5311	hsa-mir-125b-1	Unconfirmed
CID:451668	hsa-mir-219-a	Unconfirmed	CID:451668	hsa-mir-146a	24885368
CID:60750	hsa-mir-219-a	Unconfirmed	CID:3385	hsa-mir-143	19843160
CID:448537	hsa-mir-21	28265775	CID:448537	hsa-mir-221	Unconfirmed
CID:3385	hsa-mir-155	28515355	CID:3385	hsa-mir-122	24898807
CID:5311	hsa-mir-21	Unconfirmed	CID:5757	hsa-mir-34a	Unconfirmed
CID:448537	hsa-mir-155	Unconfirmed	CID:3385	hsa-let-7b	25789066
CID:5288826	hsa-mir-219-a	Unconfirmed	CID:60750	hsa-mir-146a	Unconfirmed
CID:3385	hsa-mir-146a	28466779	CID:3385	hsa-mir-1-1	Unconfirmed
CID:5757	hsa-mir-155	23568502	CID:5757	hsa-mir-20a	Unconfirmed
CID:3385	hsa-mir-125b-1	Unconfirmed	CID:3229	hsa-mir-17	Unconfirmed
CID:3385	hsa-mir-34a	25333573	CID:3385	hsa-mir-181a-1	Unconfirmed
CID:3229	hsa-mir-155	Unconfirmed	CID:5757	hsa-mir-125b-2	Unconfirmed
CID:3385	hsa-mir-125b-2	Unconfirmed	CID:3385	hsa-mir-1-2	Unconfirmed
CID:3385	hsa-mir-145	24447928	CID:448537	hsa-mir-29a	Unconfirmed
CID:3385	hsa-mir-221	27501171	CID:448537	hsa-mir-18a	Unconfirmed
CID:448537	hsa-mir-146a	Unconfirmed	CID:5757	hsa-mir-145	28011237
CID:3385	hsa-mir-126	Unconfirmed	CID:5311	hsa-mir-20a	25393367
CID:448537	hsa-mir-125b-1	Unconfirmed	CID:60750	hsa-mir-125b-1	Unconfirmed
CID:5311	hsa-mir-146a	24107356	CID:60750	hsa-mir-17	Unconfirmed
CID:3385	hsa-mir-19b-1	Unconfirmed	CID:3385	hsa-mir-223	Unconfirmed

The first column records top 1–25 related miRNAs. The second column records the top 26–50 related miRNAs

For instance, in the top 10 predicted miRNA–SM associations, the association between mir-21 and diethylstilbestrol (DES) was predicted and ranked eighth. DES is a potent synthetic estrogen and the prototypical endocrine disruptor [47]. Based on the analysis of microarray profiling data, Padmanabhan et al.’s study demonstrated that mir-21 was changed more than twofold and significantly upregulated in the samples from DES-exposed compared to control uteri [48]. The progression of the neonatal DES-induced dysplasia/neoplasia phenomenon in the hamster uterus includes a spectrum of miRNA expression alterations that differ during the initiation and promotion stages of the phenomenon [48]. These findings underscore the need for continued efforts to identify and assess both the classical genetic and the more recently recognized epigenetic mechanisms that truly drive this and other endocrine disruption phenomena [48]. What’s more, the association between mir-155 and 5-Fluorouracil (5-FU) was predicted and ranked ninth. 5-FU is a widely used chemotherapeutic drug in colorectal cancer. Using translatome profiling, a clinically relevant dose of 5-FU induces a translational reprogramming in colorectal cancer cell lines [49]. 5-FU increased the mRNA translation of HIVEP2, which encodes a transcription factor whose translation in normal condition is known to be inhibited by mir-155 [49]. In response to 5-FU, the expression of mir-155 decreases thus stimulating the translation of HIVEP2 mRNA [49]. These findings indicate that 5-FU promotes miRNA-dependent mechanisms [49].

In the top 20 predicted miRNA–SM associations, we also revealed the potential association between mir-146a and 5-FU ranked thirteenth. This association is demonstrated by Khorrami et al. [50]. In their studies, drug resistance in transfected HT-29 cells was analyzed following treatment with 5-FU [50]. The results showed overexpression of miR-146a enhanced regulatory T cells’ frequencies in peripheral blood mononuclear cells [50]. The next prediction is between mir-155 and 17β-Estradiol (E2). In estrogen responsive breast cancer cells, E2 is a key regulator of cell proliferation and survival [51]. Mir-155 is the most significantly up-regulated miRNA in breast cancer [52]. Treatment with E2 in MCF-7 cells increased miR-155 expression, promoting proliferation and decreasing apoptosis of MCF-7 cells [53]. The results demonstrated that E2 promoted breast cancer development and progression possibly through increasing the expression of miR-155 [53].

Besides, the sixteenth predicted association between mir-34a and 5-FU was verified by Li et al. [54]. Inhibition of lactate dehydrogenase A by mir-34a resensitizes colon cancer cells to 5-FU [54]. The nineteenth predicted association is between mir-145 and 5-FU. Akao’s study confirmed that the exposure to 5-FU significantly increased the intracellular levels of mir-145 in the 5-FU-sensitive human colon cancer DLD-1 cells [55]. In addition, knockdown of mir-221 in 5-FU resistant esophageal adenocarcinoma cells resulted in reduced cell proliferation, increased apoptosis, restored chemosensitivity, and led to inactivation of the Wnt/β-catenin pathway mediated by alteration in DKK2 expression [56]. The results demonstrated the association between mir-221 and 5-FU predicted by TLHNSMMA as the last in top 20.

The results in case studies have fully showed the outstanding performance of TLHNSMMA. Therefore, we further released the prediction list of the whole potential miRNAs associated with all the SMs in Dataset 1 and their association scores predicted by TLHNSMMA (see Additional file 1: Table S1).

## Discussion

MiRNAs play significant roles in the development and progression of multiple human complex diseases and discovered to be targeted by SM. Therefore, more and more attentions have focused on the identification of miRNA–SM associations in diseases, which would be helpful for developing a novel effective miRNA-associated therapeutic strategy. In this article, we integrated SM side effect similarity, SM chemical structure similarity, gene functional consistency-based similarity for SMs and miRNAs, disease phenotype-based similarity for miRNAs and SMs, disease semantic similarity, Gaussian interaction profile kernel similarity for disease, known miRNA–SM associations and known miRNA–disease associations into a triple layer network. At last, an iterative updating algorithm based on the triple layer heterogeneous graph was introduced to obtain new miRNA–SM associations. The reliable results from cross validation based on the Dataset 1 and Dataset 2 and case studies have demonstrated that TLHNSMMA could be an reliable and effective computational model for the new miRNA–SM associations identification, which would contribute to the diagnosis, treatment, prognosis, and prevention of human complex disease.

The reason of the useful performance of TLHNSMMA could be due to the following several factors. Firstly, the known experimentally confirmed miRNA–SM associations from highly reliable SM2miR v1.0 database [44] and miRNA–disease associations from reliable HMDD v2.0 database [57] used in the model for the identification of the associations between miRNAs and SMs ensured its effectiveness. Secondly, several reliable biological datasets were integrated into the heterogeneous graph. Unlike some machine learning-based model, the training data TLHNSMMA requires are only positive samples. In general, since the negative samples in machine learning-based model are randomly selected, this inaccurate chosen process would affect the model’s prediction accuracy. Therefore, the prediction accuracy of TLHNSMMA is more convincing compared with the prediction model that needs negative samples to train. Finally, global network information was used to predict potential associations between miRNAs and SMs. Compared with local network information, the advantages of global network information have been confirmed in previous researches of identifying new disease-associated genes, new disease-associated miRNA [58, 59], new disease-associated lncRNA [60] and potential drug-target interaction prediction [61]. Furthermore, TLHNSMMA took full advantage of global network information by establishing an iterative process that propagated information across the heterogeneous network, which could promote the effective prediction of TLHNSMMA. Of course, there still exist several limitations in TLHNSMMA that need to overcome in the future. TLHNSMMA cannot predict the potential SM-associated miRNAs for SMs without any known related miRNAs and potential miRNA-associated SMs for miRNAs without any known related SMs. Besides, there is no powerful approaches to obtain the optimal parameters for TLHNSMMA. Finally, the number of experimentally verified miRNA–SM associations are insufficient, there are merely 664 experimentally verified miRNA–SM associations. The more known associations between miRNA and SM need to be confirmed in the future. Although TLHNSMMA has significantly improved the prediction ability compared with previous methods, current prediction accuracy is still not satisfactory based on the evaluation of LOOCV and case studies.

## Methods

### Small molecule–miRNA associations

The miRNA–SM association dataset used in this study was acquired from the SM2miR v1.0 database [62]. The dataset contains 664 distinct experimentally confirmed miRNA–SM associations. Dataset 1 in this paper consists of 831 SMs and 541 miRNAs, only some of them are involved in the 664 known associations. Dataset 2 consists of 39 SMs and 286 miRNAs that are fully involved in the 664 known associations. Then adjacency matrix *A* is defined to represent known miRNA–SM associations. If SM s(*i*) is related to miRNA *m*(*j*), the entity *A*(*i*, *j*) is 1, otherwise 0. Furthermore, variables *ns* and *nm* are used to indicate the number of SMs and miRNAs, respectively.

### Human miRNA–disease associations

The human miRNA–disease association dataset used here was downloaded from HMDD v2.0 database [57]. In this paper, the known disease-related miRNAs that do not appear in the dataset of known miRNA–SM associations mentioned above need to be deleted. As a result, we obtained 6233 known miRNA–disease associations and established an adjacency matrix *B* to represent the known miRNA–disease associations. Similarly, variables *nd* were used as the number of diseases in the dataset, respectively. If miRNA *m*(*i*) is related to disease *d*(*j*), the entity *B*(*i*, *j*) is 1, otherwise 0.

### SM side effect similarity

We obtained SM drug side effects from SIDER [63]. Here *N*(*i*) indicates the SM *S*(*i*)-related side effect set. Based on the idea that the more side effects two SMs share, the more similar between the two SMs. If SMs have any no common side effects, their value of side effect similarity is 0. The entity $S_{S}^{s}$(*i*, *j*) used here to indicate the side effect similarity of SM *i* and SM *j*. Jaccard score [64] was used to calculate SM side effect similarity, where the notation $\left| X \right|$ is used for the cardinality of set *X*.

1 $$S_{S}^{s} = {\text{Jaccard score}} = \frac{{\left| {N\left( i \right) \cap N\left( j \right)} \right|}}{{\left| {N\left( i \right) \cup N\left( j \right)} \right|}}$$

### SM chemical structure similarity

SIMCOMP (http://www.genome.jp/tools/simcomp/) has originally been developed as a graph-based method for comparing chemical structures, which is one types of chemical structure search serves for the chemical similarity search. In this work, SIMCOMP [65] was used to calculate SM chemical structure similarities, which were collected from the DRUG and COMPOUND sections of the KEGG LIGAND database [66]. SIMCOMP is a graph-based approach of searching a maximal common sub-graph isomorphism by finding the maximal cliques in an association graph, which reflects the global score of similarity. The approach considered different environmental factors of the same atom and was widely applied to the identification of drug-target interactions. Similarly, $S_{S}^{C}$*(i*, *j)* was presented here to denote the chemical structure similarity between SM *i* and SM *j*.

### Disease phenotype-based similarity for miRNAs and SMs

miRNA–related diseases were extracted from HMDD v2.0 [57] databases miR2Disease [67] and PhenomiR [68] databases. Disease phenotype-based similarity for miRNAs was defined using the Jaccard equation [1] according to the assumption that the more diseases the miRNAs share, the more similarity between the miRNAs. Here *N*(*i*) indicates the miRNA *m*(*i*)-related disease set. The entity $S_{M}^{D} \left( {i,j} \right)$ indicated the disease phenotype-based similarity between miRNAs *i* and miRNA *j.* Similarly, SM-related diseases were extracted from Comparative Toxicogenomics Database (CTD) [69], DrugBank [70] and Therapeutic Targets Database (TTD) [71]. The entity $S_{S}^{D} \left( {i,j} \right)$ was defined here using the Jaccard score to indicate the disease phenotype-based similarity between SM *i* and SM *j*.

### Gene functional consistency-based similarity for SMs and miRNAs

We obtained the target genes of each miRNA from TargetScan [72]. Based on the assumption that if targets of two miRNAs have functional consistency, the similarity between the two miRNAs is greater. Gene Set Functional Similarity (GSFS) method [73] was used in this paper to reflect functional consistency similarity between two miRNAs by calculating functional consistency of their miRNA target gene sets [73]. The entity $S_{M}^{T} \left( {i,j} \right)$ indicates the gene functional consistency-based similarity between miRNAs *i* and miRNA *j*. Target genes of the SMs could be obtained from DrugBank and TTD. The entity $S_{S}^{T} \left( {i,j} \right)$ indicates the gene functional consistency-based similarity between SMs *i* and SM *j*.

### Integrated SM similarity

In this study, we construct integrated SM similarity based on SM side effect similarity [74], gene functional consistency-based similarity for SMs and miRNAs [75], SM chemical structure similarity [76], disease phenotype-based similarity for SMs and miRNAs [74], respectively. In order to reduce the deviation of each similarity and balance the four similarity, a weighed combination strategy was developed to integrate the similarity. As shown in Equations [2]. The integrated SM similarity $S_{S}$ can be defined as follows:

2 $$S_{S} = \left( {\beta_{1} S_{S}^{D} + \beta_{2} S_{S}^{T} + \beta_{3} S_{S}^{C} + \beta_{4} S_{S}^{s} } \right)/\mathop \sum \limits_{j} \beta_{j}\quad \left( {j = 1,2,3,4} \right)$$

Here, the default value $\beta_{j} = 1$ indicates each separated similarities have the same weight.

### Integrated miRNA similarity

Integrated miRNA similarity was established in this model by combining gene functional consistency-based similarity for miRNAs and disease phenotype-based similarity for miRNAs [74, 75]. Similarly, we used a weighed combination strategy to integrate the similarities. The integrated miRNA similarity $S_{M}$ can be defined as follows:

3 $$S_{M} = \left( {\alpha_{1} S_{M}^{D} + \alpha_{2} S_{M}^{T} } \right)/\mathop \sum \limits_{i} \alpha_{i}\quad \left( {i = 1,2} \right)$$

Here, the default value $\alpha_{i} = 1$ means each separated similarities possess the same weight.

### Disease semantic similarity model 1

Disease semantic similarity was proposed by combination of two models on the basis of disease directed acyclic graph (DAG) [77]. As illustrated in the literature [78], the semantic information of disease $d\left( i \right)$ was explained by a DAG where $d\left( i \right)$ and its ancestor diseases were used as nodes. The DAGs were retrieved from the U.S. National Library of Medicine (MeSH) at https://www.nlm.nih.gov/mesh/. The *DAG*(*D*)= (*D*, *T*(*D*), *E*(*D*)) represents the disease *D*, where *T*(*D*) is the node set of node *D* itself and its ancestor nodes, *E*(*D*) indicates the edges between child and parent nodes. We defined the contribution of disease *d* to the semantics of disease *D* as follows:

4 $$\left\{ {\begin{array}{*{20}l} {D_{D} 1\left( d \right) = 1} \hfill & {if\; d = D} \hfill \\ {D_{D} 1\left( d \right) = \hbox{max} \left\{ {\Delta *D_{D} 1\left( {d^{\prime}} \right) |d^{\prime} \in children\;of\; d} \right\} } \hfill & {if\; d \ne D} \hfill \\ \end{array} } \right.$$

Here, $\Delta$ is the semantic contribution factor and the contribution of disease *D* to the semantic value of itself is 1. Besides, the contribution of other diseases to the semantic value of disease *D* will decrease when the distance between this disease and disease *D* increases. The semantic value for disease *D* can be calculated as follows:

5 $$DV1\left( D \right) = \mathop \sum \limits_{d \in T\left( D \right)} D_{D} 1\left( d \right)$$

According to the assumption that two diseases with larger semantic similarity would share larger part of their DAGs, the value of semantic similarity between disease *d*(*i*) and *d*(*j*) in disease semantic similarity model 1 can be defined as follows:

6 $$SS1\left( {d\left( i \right),d\left( j \right)} \right) = \frac{{\mathop \sum \nolimits_{{t \in T\left( {d\left( i \right)} \right) \cap T\left( {d\left( j \right)} \right)}} (D_{d\left( i \right)} 1\left( t \right) + (D_{d\left( j \right)} 1\left( t \right))}}{{DV1\left( {d\left( i \right)} \right) + DV1\left( {d\left( j \right)} \right)}}$$

### Disease semantic similarity model 2

According to the different disease terms in the same layer of DAG (*D*) may appear in the different numbers of disease DAGs. For example, the first disease and the second disease appear in the same layer of DAG (*D*) and the first disease appears in less disease DAGs than the second disease. We can conclude that the first disease is more specific than the second disease. Therefore, the contribution of the first disease to the semantic value of disease D should be higher than the second disease. The contribution of disease in DAG to the semantic value of disease *D* can be defined as follows:

7 $$D_{D} 2\left( {\text{d}} \right) = - { \log }\left[ {\frac{The\;number\; of\;DAGs\; including\; d}{The\; number\;of\;diseases}} \right]$$

The value of semantic similarity between disease *d*(*i*) and *d*(*j*) can be calculated in disease semantic similarity model 2 as follows:

8 $${\text{SS}}2\left( {d\left( {\text{i}} \right),d\left( {\text{j}} \right)} \right) = \frac{{\mathop \sum \nolimits_{{t \in T\left( {d\left( i \right)} \right) \cap T\left( {d\left( j \right)} \right)}} (D_{d\left( i \right)} 2\left( t \right) + (D_{d\left( j \right)} 2\left( t \right))}}{{DV2\left( {d\left( i \right)} \right) + DV2\left( {d\left( j \right)} \right)}}$$

### Gaussian interaction profile kernel similarity for disease

Based on the idea that similar diseases are more likely to relate with miRNAs with similar functions. We calculate Gaussian interaction profile kernel similarity for diseases by building binary vector *IP*(*d*(*u*)) to represent the interaction profiles of disease *d*(*u*) with each miRNA, i.e. the *i*th row of the adjacency matrix *B.* Therefore, we defined Gaussian interaction profile kernel similarity between diseases *d*(*u*) and *d*(*v*) as follows.

9 $$KD\left( {d\left( u \right),d\left( v \right)} \right) = exp\left( { - \gamma_{d} \left| {\left| {IP\left( {d\left( u \right)} \right) - IP\left( {d\left( v \right)} \right)} \right|} \right|^{2} } \right)$$

where parameter $\gamma_{d}$ is used to control the kernel bandwidth, which can be obtain from the standardization of a new bandwidth $\gamma_{d}^{{\prime }}$ by the average number of related-miRNAs for per disease. Therefore, $\gamma_{d}$ can be defined as follows.

10 $$\gamma_{d} = \gamma_{d}^{{\prime }} /\left( {\frac{1}{nd}\mathop \sum \limits_{n = 1}^{nd} \left| {\left| {IP\left( {d\left( u \right)} \right)} \right|} \right|^{2} } \right)$$

### Integrated disease similarity

We introduced a Directed Acyclic Graph (DAG) to describe a disease based on the MeSH descriptors. The semantic similarity score was calculated based on the assumption that two diseases with larger shared area of their DAGs may have greater similarity score. In fact, we couldn’t get DAGs for all diseases. In other words, for the specific disease without DAG, we couldn’t calculate the semantic similarity score between the disease and other diseases. Therefore, for those disease pairs with semantic similarity score, we used the semantic similarity score to denote the disease similarity, for the others, the Gaussian interaction profile kernel similarity score was used to denote the disease similarity. Accordingly, integrated disease similarity matrix $S_{D}$ was constructed by integrating disease semantic similarity model 1, disease semantic similarity model 2 and Gaussian interaction profile kernel similarity for disease. The formulation was showed as follows:

11 $$S_{D} \left( {d\left( u \right),d\left( v \right)} \right) = \left\{ {\begin{array}{*{20}l} {\frac{{SS1\left( {d\left( u \right),d\left( v \right)} \right) + SS2\left( {d\left( u \right),d\left( v \right)} \right)}}{2} } \hfill & \quad {d\left( u \right)\; and\; d\left( v \right)\; has\; functional\; similarity} \hfill \\ {KD\left( {d\left( u \right),d\left( v \right)} \right)} \hfill &\quad {otherwise} \hfill \\ \end{array} } \right.$$

### TLHNSMMA

**Fig. 4:**
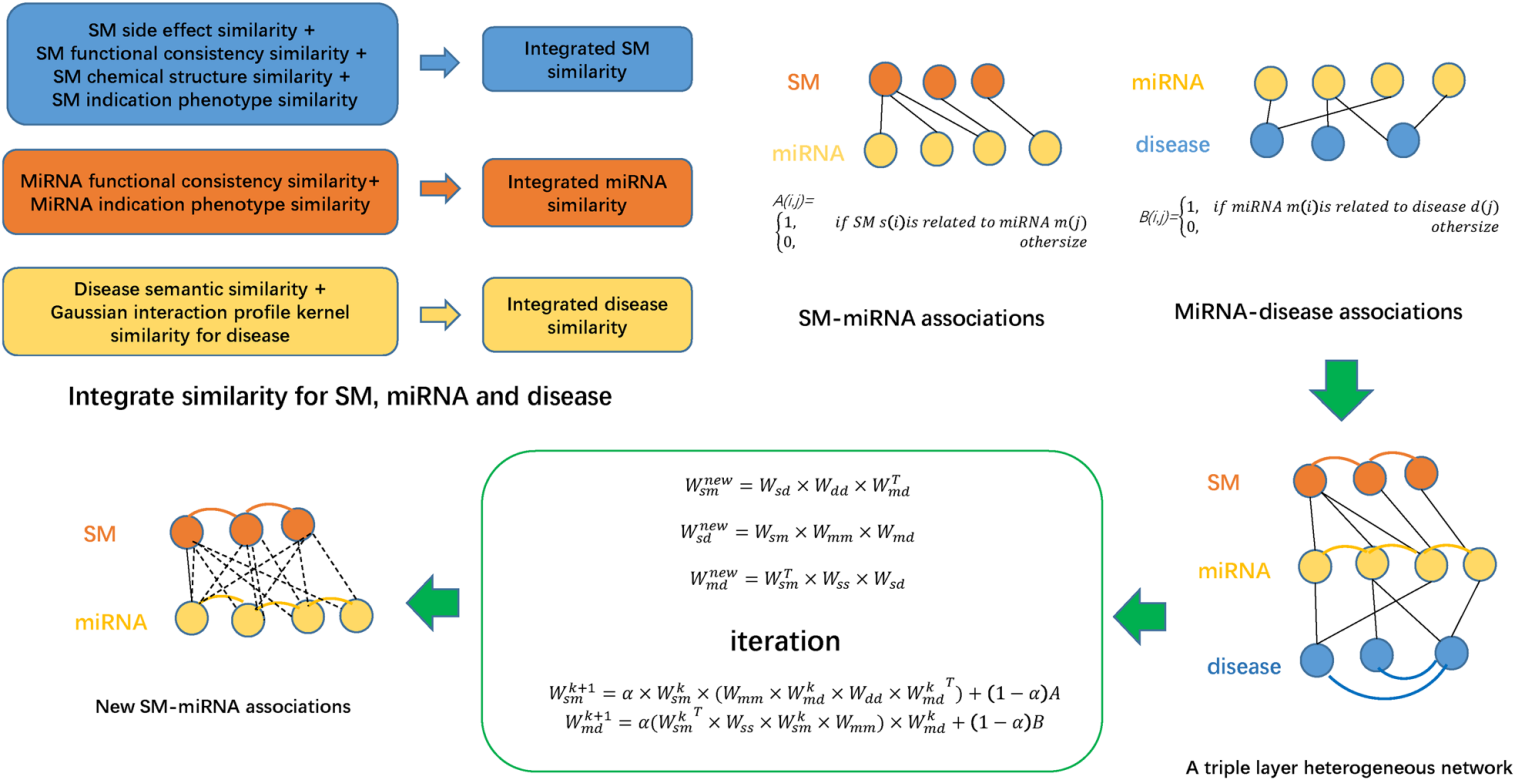
Flowchart of TLHNSMMA model to predict the potential miRNA–SM associations based on the known associations in SM2miR V1.0 database

Based on the guilt-by-association principle [79, 80], potential miRNA–SM associations can be predicted by constructing two-layer heterogeneous network with the datasets of integrated miRNA similarity, integrated SM similarity and known miRNA–SM associations. Likewise, novel miRNA–disease associations can be inferred by constructing two-layer heterogeneous network with the datasets of integrated miRNA similarity, integrated disease similarity and known miRNA–disease associations. Therefore, here we infer potential miRNA–SM associations in the newly developed three-layer model by integration of known miRNA–SM associations, miRNA–disease associations, integrated similarity for SMs, miRNAs and diseases using an information flow-based method (see Fig. 4). To establish new associations between SMs and diseases that have no associations originally, we can calculate a new $W_{sd}^{new}$ in matrix format as follow:

12 $$W_{sd}^{new} = W_{sm} \times S_{M} \times W_{md}$$

which incorporates integrated miRNAs similarity $S_{M} ,$ miRNA–SM associations $W_{sm}$ and miRNA–disease associations $W_{md}$. According to the association between SMs and diseases established above, new associations between SMs and miRNAs $W_{sm}^{new}$ can be constructed using SM-disease associations $W_{sd} ,$ integrated disease similarity $S_{D}$ and miRNA–disease associations $W_{md}$.

13 $$W_{sm}^{new} = W_{sd} \times S_{D} \times W_{md}^{T}$$

The equation was established to infer new associations between miRNAs and SMs by consideration of diseases. What’s more, new associations between miRNA and disease $W_{md}^{new}$ can be obtained simultaneously by incorporating SM information, which can be written as follows:

14 $$W_{md}^{new} = W_{sm}^{T} \times S_{S} \times W_{sd}$$

where $S_{D}$ represents integrated disease similarity and superscript T indicates the transpose of the corresponding matrix. We set $W_{sd}^{new}$ as a temporary value and replace it in the right sides of the Eqs. (13) and (14). Then, Eqs. (13) and (14) can be rewritten as follows:

15 $$W_{sm}^{new} = W_{sm} \times S_{M} \times W_{md} \times S_{D} \times W_{md}^{T}$$

16 $$W_{md}^{new} = W_{sm}^{T} \times S_{S} \times W_{sm} \times S_{M} \times W_{md}$$

In view of the above-mentioned formula, Eqs. (15) and (16) incorporates all diseases related to miRNA and SM, as well as their similarity and all SMs related to miRNA and disease, as well as their similarity, respectively. More importantly, Eq. (15) is potentially more powerful in predicting unobserved miRNA–SM associations by consideration of the information of diseases. The new associations between SMs and diseases as a by-product in the model can be predicted using Eq. (12). The same is true for Eq. (16). Once the new miRNA–SM associations $W_{sm}^{new}$ and new miRNA–disease associations $W_{md}^{new}$ were obtained, we could build an iterative updating procedure. To integrate the initial associations between miRNA and SM associations and initial associations between miRNA and disease associations into those predicted procedures, the final model can be built as follows:

17 $$W_{sm}^{k + 1} = \alpha \times W_{sm}^{k} \times \left( {S_{M} \times W_{md} \times S_{D} \times W_{md}^{kT} } \right) + \left( {1 - \alpha } \right)A$$

18 $$W_{md}^{k + 1} = \alpha \times \left( {W_{sm}^{kT} \times S_{S} \times W_{sm}^{k} \times S_{M} } \right) \times W_{md}^{k} + \left( {1 - \alpha } \right)B$$

where $\upalpha$ is a decay factor in the range of (0,1); *A* is the adjacency matrix of known miRNA–SM associations acquired from the SM2miR v1.0 database, defined *A*(*i*, *j*)= 1 if SM *s*(*i*) is linked with miRNA *m*(*j*) otherwise 0. B is the adjacency matrix of known miRNA–disease associations downloaded from HMDD v2.0, defined *B*(*i*, *j*)= *1* if miRNA *m*(*i*) is linked with disease *d*(*j*)*n* otherwise 0. In each iteration, the known miRNA–SM association matrix *A* and miRNA–disease association matrix *B* will contribute to the newly constructed interactions of $W_{sm}^{k}$ and $W_{md}^{k + 1}$. The contribution is controlled by the scale factor $1 - \alpha$, where $\alpha$ is a decay factor. We chose the same decay factor $\alpha$ (0.4) as the one in [81], which used the same triple layer heterogeneous network in their study, so the original known associations have slightly more weights. The associations between a miRNA and SM will finally include all the possible paths connecting them in the constructed triple layer heterogeneous network by iteratively using formula [17]. The same is true for the new miRNA–disease associations using formula [18]. These two iterative update equations can be treated as simulating a process in which each node with prior information propagates the information obtained in the previous iteration to its neighbors. Due to the relation between the end-points and the probability of looking into an edge among the same end-points in a random network with the same node degrees, the weight of an edge was normalized according to the degrees of its end-points. The two iterative update equations will converge with proper normalization, which is summarized as a theorem [82]. They will be stable after some steps and final probability scores of potential miRNA–SM associations and miRNA–disease associations will be obtained (when the change value between $W_{sm}^{k + 1}$ and $W_{sm}^{k}$ measured by L1 norm is less than a given cutoff, here the cutoff is set as 10^−6^). $W_{sm}^{k}$ and $W_{md}^{k}$ defined in Eqs. (17) and (18) will converge after proper normalization (the proof can be found in the Additional file 2).

## Additional files

**Additional file 1: Table S1**. We applied TLHNSMMA to prioritize all the candidate SM–miRNA pairs from highly reliable SM2miR V1.0 database as training samples. This prediction result is released for further experimental validation and research.

**Additional file 2.** We give the proof of THEOREM: $W_{sm}^{k}$ and $W_{md}^{k}$ defined in Eqs. (17) and (18) will converge after proper normalization.

## Abbreviations

TLHNSMMA: triple layer heterogeneous network based small molecule-MiRNA association prediction
miRNAs: microRNAs
SM: small molecule
HCV: hepatitis C virus
SMiR-NBI: small molecule–miRNA network-based inference
NBI: network based inference
SMirN: small molecule-miRNA network
SmiRN-AD: small molecule and miRNA association network in Alzheimer’s disease
LOOCV: leave-one-out cross validation
ROC: receiver operating characteristics
AUC: area under the ROC curve
DES: diethylstilbestrol
5-FU: 5-Fluorouracil
E2: 17β-Estradiol
CTD: comparative toxicogenomics database
TTD: therapeutic targets database
GSFS: gene set functional similarity
DAG: directed acyclic graph
