# Supplementary material for: Inferring potential small molecule–miRNA association based on triple layer heterogeneous network
Source: J Cheminform. 2018 Jun 26;10:30. doi: 10.1186/s13321-018-0284-9 (PMC6020102; doi:10.1186/s13321-018-0284-9)
Supplement: Supplementary file 2 — Additional file 2. We give the proof of THEOREM: $W_{sm}^{k}$Wsmk and $W_{md}^{k}$Wmdk defined in Eqs. (17) and (18) will converge after proper normalization. [file 13321_2018_284_MOESM2_ESM.docx]

**Inferring potential small molecule-miRNA association based on triple layer heterogeneous network**

Jia Qu^1^, Xing Chen^1, *^, Ya-Zhou Sun^2,3^, Jian-Qiang Li^2,3^, Zhong Ming^2,3^

^1^School of Information and Control Engineering, China University of Mining and Technology, Xuzhou, 221116, China

^2^National Engineering Laboratory for Big Data System Computing Technology, Shenzhen University, Shenzhen, 518060, China

^3^College of Computer Science and Software Engineering, Shenzhen University, Shenzhen, 518060, China

* Corresponding to Xing Chen, School of Information and Control Engineering, China University of Mining and Technology, Xuzhou, 221116, China, E-mail: xingchen@amss.ac.cn

E-mail:

Jia Qu: [TB17060015B4@cumt.edu.cn](mailto:TB17060015B4@cumt.edu.cn)

Xing Chen: [xingchen@amss.ac.cn](mailto:xingchen@amss.ac.cn)

Ya-Zhou Sun: [workdatasyz@126.com](mailto:workdatasyz@126.com)

Jian-Qiang Li: [lijq@szu.edu.cn](mailto:lijq@szu.edu.cn)

Zhong Ming: [mingz@szu.edu.cn](mailto:mingz@szu.edu.cn)

**THEOREM.** $W_{sm}^{k}$ and $W_{md}^{k}$ defined in Equations (17) and (18) will converge after proper normalization.

**PROOF of THEOREM**

For a matrix $A={(a_{i,j})}_{n\times m}$ define function *normalize(),* which takes matrix *A* as input and apply the following transformation to all its elements:$a_{i,j}=\frac{a_{i,j}}{\sqrt{\sum_{k=1}^{m} a_{i,k}}\sqrt{\sum_{k=1}^{n} a_{k,j}}}$.

Let

$f(W_{md})=normalize(S_{M}\times W_{md}\times S_{D}\times{W_{md}^{k}}^{T})$

$f(W_{sm})=normalize({W_{sm}^{k}}^{T}\times S_{S}\times W_{sm}^{k}\times S_{M})$

Then equation 17 and 18 can be rewritten as:

$$W_{sm}^{k+1}=\alpha W_{sm}^{k}\times f(W_{md}^{k})+\left( 1-\alpha\right)A$$

$W_{md}^{k+1}=\alpha f\left( W_{sm}^{k} \right)\times W_{md}^{k}+\left( 1-\alpha\right)B$

Suppose the transformation step used in the proof of (Wang, et al., 2013) as$G$.

$$W_{sm}^{{k+1}^{*}}=\alpha{G(I,f(W_{md}^{k}))W}_{sm}^{k^{*}}+\left( 1-\alpha\right)A^{*}$$

$W_{md}^{{k+1}^{*}}=\alpha G(f\left( W_{sm}^{k} \right),I)W_{md}^{k^{*}}+\left( 1-\alpha\right)B^{*}$

$\left[ {W_{sm}^{{k+1}^{*}} \atop W_{md}^{{k+1}^{*}}} \right] =\alpha\left[ \begin{matrix} G\left( I,f\left( W_{md}^{k} \right) \right), & 0 \\ 0, & G(f\left( W_{sm}^{k} \right),I) \end{matrix} \right]\left[ \begin{matrix} W_{sm}^{k^{*}} \\ W_{md}^{k^{*}} \end{matrix} \right]$+$\left( 1-\alpha\right)\left[ \begin{matrix} A^{*} \\ B^{*} \end{matrix} \right]$

The eigenvalue of $\left[ \begin{matrix} G\left( I,f\left( W_{md}^{k} \right) \right), & 0 \\ 0, & G(f\left( W_{sm}^{k} \right),I) \end{matrix} \right]$ is in the range of $[-1,1]$. Therefore, we have$\left\| \begin{matrix} W_{sm}^{k^{*}} \\ W_{md}^{k^{*}} \end{matrix} \right\|\leq\left\| \begin{matrix} A^{*} \\ B^{*} \end{matrix} \right\|$

We must prove that $H(\left[ \begin{matrix} X_{1} \\ X_{2} \end{matrix} \right]) =\alpha\left[ \begin{matrix} G\left( I,f\left( X_{2} \right) \right), & 0 \\ 0, & G(f\left( X_{1} \right),I) \end{matrix} \right]\left[ \begin{matrix} X_{1} \\ X_{2} \end{matrix} \right]$+$\left( 1-\alpha\right)\left[ \begin{matrix} X_{1}^{0} \\ X_{2}^{0} \end{matrix} \right]$ is a contraction mapping on the domain$\left\| X \right\|\leq\left\| X^{0} \right\|$. Apparently, for any given *X,* we have $\left\| H(X) \right\|\leq\left\| X^{0} \right\|$. Because $\left\| G\left( I,f\left( X \right) \right) \right\|$ is continuous, for any pair of vector $\left[ \begin{matrix} X_{11} \\ X_{12} \end{matrix} \right]$ and $\left[ \begin{matrix} X_{21} \\ X_{22} \end{matrix} \right]$ in the domain $\left\| X \right\|\leq\left\| X^{0} \right\|$, there is a $\lambda>0$.

$$\left\| \left[ \begin{matrix} G\left( I,f\left( X_{12} \right) \right)-G\left( I,f\left( X_{22} \right) \right), & 0 \\ 0, & G\left( f\left( X_{11} \right),I \right)-G\left( f\left( X_{21} \right),I \right) \end{matrix} \right] \right\|<\lambda\left\| \left[ \begin{matrix} X_{11} \\ X_{12} \end{matrix} \right]-\left[ \begin{matrix} X_{21} \\ X_{22} \end{matrix} \right] \right\|$$

$$\left\| H(\left[ \begin{matrix} X_{11} \\ X_{12} \end{matrix} \right])-H(\left[ \begin{matrix} X_{21} \\ X_{22} \end{matrix} \right]) \right\|=\alpha\left\| \left[ \begin{matrix} G\left( I,f\left( X_{12} \right) \right), & 0 \\ 0, & G(f\left( X_{11} \right),I) \end{matrix} \right]\left[ \begin{matrix} X_{11} \\ X_{12} \end{matrix} \right]-\left[ \begin{matrix} G\left( I,f\left( X_{22} \right) \right), & 0 \\ 0, & G(f\left( X_{21} \right),I) \end{matrix} \right]\left[ \begin{matrix} X_{21} \\ X_{22} \end{matrix} \right] \right\|$$

$\mathbf{=}\alpha\left\| \left[ \begin{matrix} G\left( I,f\left( X_{12} \right) \right), & 0 \\ 0, & G(f\left( X_{11} \right),I) \end{matrix} \right]\left[ \begin{matrix} X_{11}-X_{21} \\ X_{12}-X_{22} \end{matrix} \right]+ \left[ \begin{matrix} G\left( I,f\left( X_{12} \right) \right)-G\left( I,f\left( X_{22} \right) \right), & 0 \\ 0, & G\left( f\left( X_{11} \right),I \right)-G(f\left( X_{21} \right),I) \end{matrix} \right]\left[ \begin{matrix} X_{21} \\ X_{22} \end{matrix} \right] \right\|< \alpha\left\| \left[ \begin{matrix} X_{11}-X_{21} \\ X_{12}-X_{22} \end{matrix} \right] \right\|$+$\alpha\lambda\left\| \left[ \begin{matrix} X_{11}-X_{21} \\ X_{12}-X_{22} \end{matrix} \right] \right\|\left\| X^{0} \right\|$

=$\alpha(1+\lambda\left\| X^{0} \right\|)\left\| \left[ \begin{matrix} X_{11}-X_{21} \\ X_{12}-X_{22} \end{matrix} \right] \right\|$

Once we have $\left\| X^{0} \right\|$ small enough, we can have

$\left\| H(\left[ \begin{matrix} X_{11} \\ X_{12} \end{matrix} \right])-H(\left[ \begin{matrix} X_{21} \\ X_{22} \end{matrix} \right]) \right\|<\left\| \left[ \begin{matrix} X_{11}-X_{21} \\ X_{12}-X_{22} \end{matrix} \right] \right\|$. Therefore *H* is a contraction mapping and there is a fixed point for *H* in $\left\| (X) \right\|\leq\left\| X^{0} \right\|$. In fact, in our calculation, we have *H* converged without any normalization on $X^{0}$.
